# Use of Femtosecond Laser for Cataract Surgery in Patients With Previous Anterior Lamellar Keratoplasty and Corneal Scar: A Case Report

**DOI:** 10.1155/crop/3238383

**Published:** 2026-07-22

**Authors:** Jordan J. Huang, Yordan Urrutia, Jose Miguel Mora Correa, Allister Gibbons, Jaime D. Martinez

**Affiliations:** ^1^ Bascom Palmer Eye Institute, University of Miami, Miami, Florida, USA, miami.edu; ^2^ The Ottawa Hospital, University of Ottawa Eye Institute, Ottawa, ON, Canada, uottawa.ca; ^3^ Florida State University College of Medicine, Tallahassee, Florida, USA, fsu.edu

**Keywords:** anterior lamellar keratoplasty, case report, cataract, corneal scar, femtosecond laser

## Abstract

This study describes the outcomes of femtosecond laser‐assisted cataract surgery (FLACS) in patients with prior corneal scarring and prior anterior lamellar keratoplasty (ALK). Two male patients, aged 65 and 61 years, presented for cataract surgery with a central corneal scar and previous ALK, respectively. Both underwent standard FLACS to address their cataracts amidst these complex corneal conditions. The procedure successfully achieved a centered 5.0 mm capsulotomy without tags or tears, and the lens was segmented into a sextant pattern and removed without difficulty. No serious intraoperative complications occurred. In the first case, best corrected visual acuity improved from 20/150 to 20/40 at 1‐month postoperation, with the patient reporting enhanced visual clarity. In the second case, visual acuity improved from 20/800 to 20/100 1 month after surgery, with further enhancement to 20/60 following scleral lens fitting. This study represents an existing technique on the use of FLACS in patients with ALK and corneal scarring. The findings demonstrate that FLACS can be effectively and safely performed in these challenging cases, providing valuable insights into its potential advantages over traditional manual cataract surgery, particularly in patients with compromised ocular structures and limited lens visibility.

## 1. Introduction

Femtosecond Laser‐assisted cataract surgery (FLACS) is an established surgical technology that uses ultrashort laser pulses to create cleavage planes via photodisruption of transparent/translucent tissues [1]. Coupled with live optical coherence tomography (OCT) imaging, it has become commonly utilized during cataract surgery for corneal incisions, capsulotomy, and lens fragmentation [1, 2]. Compared to conventional cataract surgery, the use of a femtosecond laser has been purported to potentially improve surgical precision, patient safety, and time efficiency [1]. In patients with complicated ocular histories, such as patients with corneal scars, lens capsule damage, traumatic cataract, corneal penetrating injuries, zonular compromise, or intumescent cataracts, manual capsulotomy can be difficult, largely due to hindered visibility and fragility of ocular structures [2–5]. Thus, FLACS may serve as an important adjunct for difficult cataract extractions. However, patients with corneal opacities have been traditionally excluded from FLACS due to concerns about incomplete laser penetration, potentially leading to capsular tags, and issues with lens fragmentation [2–5].

To our knowledge, no previous reports exist on the use of FLACS in a patient with previous anterior lamellar keratoplasty (ALK), and few reports exist on FLACS in patients with corneal scars [2–5]. As such, the purpose of this case report is to detail the successful use of FLACS in two patients, one with a previous ALK and one with a corneal scar.

## 2. Case Reports

### 2.1. Case 1

**Figure 1:**
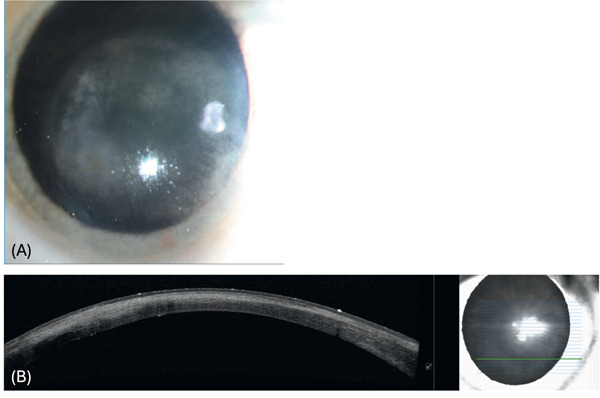
(A) Slit lamp of the left eye examination identified a central and inferior corneal scar, inferior ghost vessels, and a mixed cataract consisting of nuclear sclerosis, cortical cataract, and posterior subcapsular cataract. (B) OCT showing inferior cornea thinning with anterior stromal hyperreflectivity.

A 65‐year‐old man presented for cataract evaluation of his left eye following complaints of gradual worsening distance vision and blurriness over the past few years. Previous ocular history was significant for infectious keratitis secondary to contact lens wear in his left eye. He had no previous ocular surgeries. At the time of evaluation, best spectacle‐corrected distance visual acuity was 20/150, with improvement on pinhole to 20/60 and potential acuity measured as 20/40 in his left eye. He was fitted for scleral lenses, which led to improvements in his distance visual acuity to 20/60. Slit lamp exam revealed a central and inferior corneal scar, inferior ghost vessels, and a mixed cataract comprised of a nuclear sclerotic cataract with cortical cataract and a posterior subcapsular cataract. Using the modified Fantes haze scale, the corneal haziness was classified as Grade 2 (Figure 1A). Preoperative OCT anterior segment imaging revealed inferior corneal thinning with anterior stromal hyperreflectivity between the 10% and 60% depth (Figure 1B). The posterior pole was adequately examined despite the media opacity, and no pathologic changes were identified. The use of the femtosecond laser was discussed, and the patient elected to proceed with FLACS. Standard FLACS was performed using a femtosecond laser Catalys Precision Laser System (Johnson & Johnson Surgical Vision, Inc., Irvine, California, United States).

**Figure 2:**
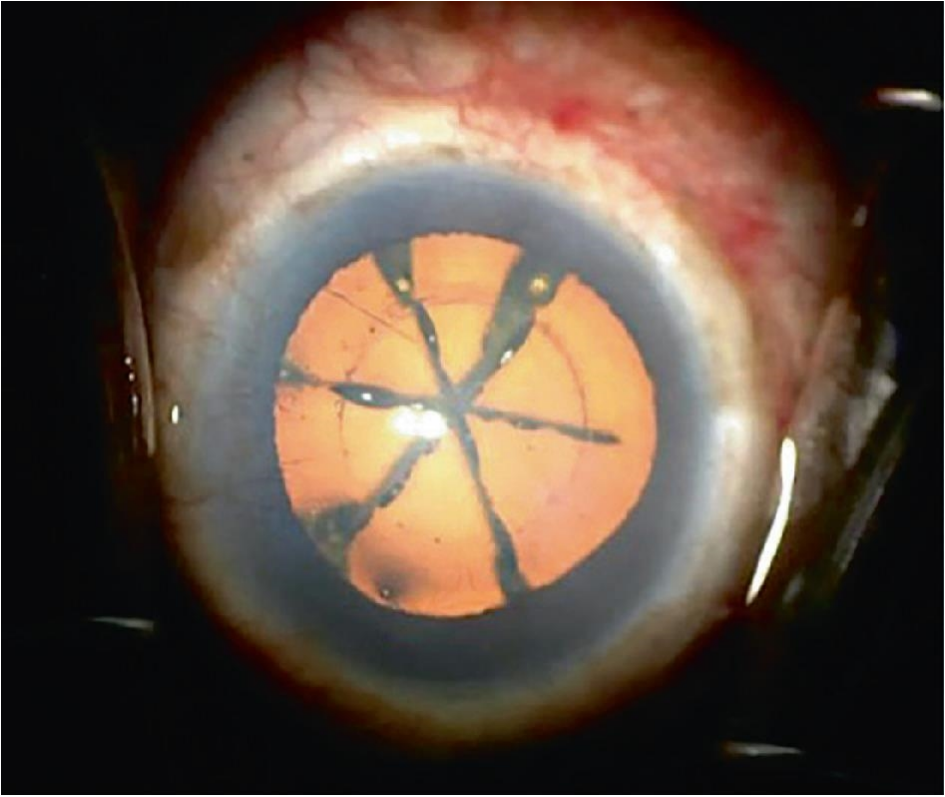
Intraoperative microscope photograph of Case 1 obtained in retroillumination mode, demonstrating the sextant lens fragmentation pattern and femtosecond laser capsulotomy.

The capsulotomy pulse energy was maximized to 4.0 *μ*J, and depth was set at 400 *μ*m. A standard 5.0 mm capsulotomy was performed by the laser with a sextant pattern lens fragmentation (Figure 2). There were no complications with the capsulotomy, which appeared round, centered, and without tags or tears. Following this, the patient underwent standard cataract surgery. The operating surgeon had no difficulties removing the prefragmented wedges via retroillumination on the surgical microscope. A monofocal lens was then inserted. Postoperative visual acuity at the 1‐week time point was 20/200 with pinhole to 20/40. At 1‐month postoperation, visual acuity improved to 20/150 with pinhole to 20/40, with the patient reporting improved visual clarity. The patient will undergo scleral refitting for further improvement of visual acuity.

### 2.2. Case 2

**Figure 3:**
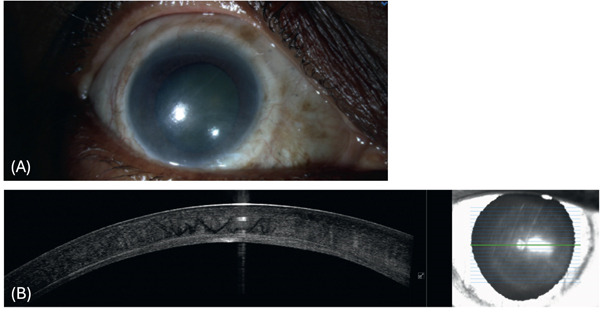
(A) Slit lamp photos depicting corneal scarring with neovascularization. (B) Preoperative OCT image showing anterior lamellar keratoplasty with stromal bed and a zigzag stromal pattern on the interface.

A 61‐year‐old man presented to our service with increasing blurriness in his right eye. At the time of evaluation, distance visual acuity was 20/800 with no improvement on spectacle refraction or pinhole to the right eye and 20/25 in his left eye. Previous ocular history was significant for interstitial keratitis of presumed herpetic origin OD, of which he was treated with antivirals and prednisolone eye drops. The patient ultimately underwent an ALK without complications and had no recurrence of the corneal infection. Slit lamp exam revealed a nuclear sclerotic cataract, an intact corneal graft with mild stromal haziness likely secondary to the stromal donor interface, and corneal neovascularization at the 3 o′clock position with no lipid deposition. Using the modified Fantes haze scale, the corneal haziness was classified as Grade 3 (Figure 3A). Preoperative OCT anterior segment imaging showed the ALK with stromal bed and a zigzag stromal pattern on the interface (Figure 3B). Standard FLACS was performed using a femtosecond laser Catalys Precision Laser System (Johnson & Johnson Surgical Vision, Inc., Irvine, California, United States), under the same settings as Case 1.

**Figure 4:**
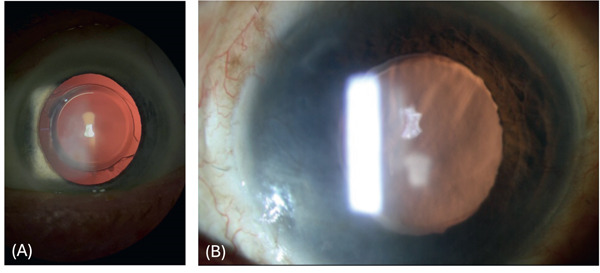
Postoperative 1‐month slit lamp photos for Case 1 and Case 2. (A) Case 1 photos showing a well‐centered intraocular lens on retroillumination. (B) Case 2 photos showing a well‐centered intraocular lens on retroillumination.

The laser was used to perform a 5.0 mm capsulotomy followed by a sextant pattern lens fragmentation. No complications occurred. Following this, the patient underwent standard cataract surgery in which a standard monofocal lens was inserted. In addition, the corneal neovascularization was cauterized, and 1.25 mg in 0.05 mL of bevacizumab was injected into the subconjunctiva. There were no intraoperative complications. Two tags were noted during capsulotomy removal; however, they were easily managed and removed without tears or extension, or any further complication. At the 1‐month postoperative visit, uncorrected visual acuity improved to 20/150 and pinhole to 20/100. Scleral lens fitting resulted in further visual acuity improvement to 20/60. One‐month postoperative slit lamp photos are provided for Case 1 and Case 2 (Figure 4).

## 3. Discussion

To date, few studies exist on the use of a femtosecond laser for cataract surgery in complicated ocular cases such as those involving patients with corneal scarring, lens capsule damage, corneal penetrating injuries, zonule damage, or white traumatic cataract [2–5]. Grewal et al. found success in the use of a femtosecond laser Catalys Precision Laser System (Johnson & Johnson Surgical Vision, Inc., Irvine, California, United States) in cataract surgery for a patient with a history of traumatic corneal scar and cataracts. They detail a technique to customize the femtosecond laser and OCT to laser and image through paracentral and central corneal scars, although a smaller, 3.7 mm capsulotomy was achieved due to poor dilation [2]. Conrad‐Hengerer et al. report the use of the femtosecond laser Catalys Precision Laser System (Johnson & Johnson Surgical Vision, Inc., Irvine, California, United States) for a patient with a history of a penetrating injury to the cornea and lens capsule. They found the use of the femtosecond laser minimized damage to the capsular bag and zonular apparatus after treatment [3]. Nagy et al. achieved success in the use of a femtosecond laser Alcon (LenSx Inc Aliso Viejo, California, United States) in a traumatic cataract following penetrating eye injury, an anterior capsule laceration, and a blunt trauma resulting in white cataracts [4].

Additionally, very few reports exist in the literature on the use of femtosecond laser for cataract surgery in patients with a history of keratoplasty [6–9]. Awidi et al. described a case of a 54‐year‐old female with a history of penetrating keratoplasty (PK) complicated by endophthalmitis needing pars plana vitrectomy leading to rapid cataract progression [6]. Femtosecond laser was utilized to create arcuate incisions, 5 mm capsulotomy, and lens softening. They concluded that FLACS was reliable and safe in treating complex cataract cases with a history of PK. Cao et al. described a case of a 61‐year‐old male with a 2‐year history of 7 mm PK in the left eye that presented with a dense cataract with a 3 mm centered white anterior lens capsule calcification [7]. The LenSx laser system (Alcon LenSx Inc, Aliso Viejo, California, United States) was utilized, and a 5 mm capsulotomy was performed. An AS‐OCT‐guided 2.2 mm corneal incision was created, and a chop pattern was used for lens fragmentation. They concluded that utilizing femtosecond laser technology enables accurate incisions, controlled capsulorhexis, and diminishes the need for ultrasound energy during lens removal. Their approach minimized potential complications in cataract surgery following PK, leading to enhanced visual recovery and improved refractive outcomes.

Tolees et al. described two patients with a history of deep anterior lamellar keratoplasty (DALK) that developed a significant cataract and subsequently underwent FLACS [8]. The first patient was a 59‐year‐old male with a history of DALK in the right eye for advanced keratoconus. FLACS (Alcon LenSx Inc, Aliso Viejo, California, United States) was performed with a 4.8 mm capsulorhexis, nuclear fragmentation, and three corneal incisions, a single 2.8‐mm two‐plane main incision and two 1.0 mm single‐plane side‐port incisions. The second patient was a 27‐year‐old male 9‐month status‐post DALK of the right eye with posterior subcapsular cataract. The FLACS procedure was carried out with the same parameters as the first case, except the angle of the side ports was changed from 70° to 30°. FLACS can offer extreme precision during the creation of the corneal incision and capsulorhexis, which may result in an alternative technique in cataract surgery after DALK surgery. Interestingly, even though our patient had a thicker stromal bed due to a previous anterior keratoplasty, this did not impact the ability of the laser to perform the capsulorhexis and fragmentation, although further studies are required to ensure safety. Although visualization was suboptimal, the subsequent removal of the fragmented lens was notably less challenging and less taxing for the surgeon.

Corneal scars may alter laser‐tissue interaction, leading to irregular photodisruption patterns. For example, dense scars can absorb laser energy, reducing effective capsulotomy completion and necessitating careful capsulotomy removal. In cases of significant scar burden, increasing laser energy or spot density in affected zones may improve capsulotomy continuity, though this risks collateral tissue damage [9]. Further studies are needed to optimize safety profiles.

Previous studies on FLACS for corneal scars have utilized a pulse energy of 10 *μ*J for capsulotomy [2, 3]. This is based on a previous literature report on a 10‐year‐old boy with ectopia lentis due to Marfan′s syndrome, in which FLACS was performed using 10 *μ*J of pulse energy for the capsulotomy [10]. This patient did not have a corneal scar. To date, we have had success with using standard FLACS settings at a pulse energy of 4 *μ*J without any complications. However, we recognize that dense corneal scars may require setting customization of the femtosecond laser, such as by increasing the pulse energy setting, in order to effectively perform both capsulotomy and lens fragmentation.

However, corneal scars may disrupt the laser′s imaging system by scattering light, leading to incomplete or distorted 3D reconstructions of the anterior capsule and lens. OCT relies on a technique known as interferometry to measure the optical path length of light returning from the sample. As a result, OCT can generate clear, three‐dimensional images of thick tissues by suppressing background noise. It also enables real‐time imaging beneath the surface, offering immediate visualization of structures [11]. LenSx and Catalys incorporate spectral‐domain OCT imaging, while comparing to Victus (Bausch + Lomb), laser uses real‐time streaming swept‐source OCT and the Lensar Laser System (Lensar) uses Scheimpflug‐guided imaging.

In contrast, the Scheimpflug principle captures images of the anterior segment of the eye by positioning a camera at an angle to a slit beam, forming an optical cross‐section of the cornea and lens. Scheimpflug imaging provides precise and repeatable measurements of parameters such as central corneal thickness and anterior chamber depth. However, due to optical refraction and the angular camera setup, the raw images are inherently distorted, particularly exhibiting compression perpendicular to the optical axis [12]. Scheimpflug imaging may offer improved penetration at the cost of reduced resolution. Further comparative studies between systems should be conducted.

In this study, we report the usage of FLACS technique in a patient with previous ALK surgery. In our study, we were able to successfully perform a capsulotomy and lens fragmentation in one patient with a previous ALK surgery and one patient with a corneal scar, under standard femtosecond laser settings. We did not encounter any incomplete capsulotomies or fragmentations. We found the use of retroillumination mode along with OCT during FLACS greatly aided in our ability to visualize the cataract throughout the procedure. The results of our study detail the safe and effective use of a FLACS in patients with previous ALK and corneal scarring. We highlight the usage of FLACS as an alternative in cases with increased fragility of ocular structures, or limited ability to visualize the lens.

## 4. Limitations

This report is limited by the small number of cases and a short follow‐up period. Because only two patients were included, the results should be interpreted cautiously and cannot be generalized to all eyes with corneal scarring or prior ALK. Larger studies with longer follow‐up are needed to confirm the safety and reproducibility of this approach.

## Author Contributions

J.J.H.: conceptualization, data curation, formal analysis, and writing—original draft. Y.U.: data curation, investigation, and writing—review and editing. J.M.M.C.: investigation, literature review, and writing—review and editing. A.G.: supervision, validation, and writing—review and editing. J.D.M.: conceptualization, supervision, project administration, and writing—review and editing.

## Funding

The study was supported by NIH Center Core (P30EY014801) and Research to Prevent Blindness (10.13039/100001818, GR004596).

## Disclosure

All authors attest that they meet the current ICMJE criteria for authorship.

## Consent

Written informed consent was obtained from both patients for publication of this case report and the accompanying images. All research was conducted ethically in accordance with the Declaration of Helsinki.

## Conflicts of Interest

The authors declare no conflicts of interest.

## Data Availability

The data are not publicly available due to patient privacy and ethical restrictions.

## References

[1] Sun H. , Fritz A. , Droge G. , Neuhann T. , and Bille J. F. , Femtosecond-Laser-Assisted Cataract Surgery (FLACS), High Resolution Imaging in Microscopy and Ophthalmology: New Frontiers in Biomedical Optics, 2019, Springer, 10.1007/978-3-030-16638-0_14. 32091677

[2] Grewal D. S. , Basti S. , and Grewal S. P. S. , Customizing Femtosecond Laser-Assisted Cataract Surgery in a Patient With a Traumatic Corneal Scar and Cataract, Journal of Cataract & Refractive Surgery. (2014) 40, no. 11, 1926–1927, 10.1016/j.jcrs.2014.09.021, 25442886. 25442886

[3] Conrad-Hengerer I. , Dick H. B. , Schultz T. , and Hengerer F. H. , Femtosecond Laser-Assisted Capsulotomy After Penetrating Injury of the Cornea and Lens Capsule, Journal of Cataract & Refractive Surgery. (2014) 40, no. 1, 153–156, 10.1016/j.jcrs.2013.11.001, 24355728. 24355728

[4] Nagy Z. Z. , Kranitz K. , Takacs A. , Filkorn T. , Gergely R. , and Knorz M. C. , Intraocular Femtosecond Laser Use in Traumatic Cataracts Following Penetrating and Blunt Trauma, Journal of Refractive Surgery. (2012) 28, no. 2, 151–153, 10.3928/1081597X-20120120-01, 22313435. 22313435

[5] Szepessy Z. , Takacs A. , Kranitz K. , Filkorn T. , and Nagy Z. , Intraocular Femtosecond Laser Use in Traumatic Cataract, European Journal of Ophthalmology. (2014) 24, no. 4, 623–625, 10.5301/ejo.5000443, 24519509. 24519509

[6] Awidi A. , Dzhaber D. , and Daoud Y. J. , Application of Femtosecond Laser-Assisted Cataract Surgery in Patients With Corneal Pathologies, American Journal of Ophthalmology Case Reports. (2018) 11, 170–171, 10.1016/j.ajoc.2018.06.015, 30128369. 30128369 PMC6097640

[7] Cao D. , Wang S. , and Wang Y. , Femtosecond Laser-Assisted Cataract Surgery After Penetrating Keratoplasty: A Case Report, BMC Ophthalmology. (2017) 17, no. 1, 1–4, 10.1186/s12886-017-0496-1. 28646864 PMC5483306

[8] Tolees S. S. , El-Danasoury A. M. , and Hashem A. , Femtosecond Laser Assisted Cataract Surgery After Deep Lamellar Keratoplasty: Case Report, Journal of Clinical and Experimental Ophthalmology. (2014) 5, no. 4, 1–3, 10.4172/2155-9570.1000348.

[9] Schultz T. , Ezeanosike E. , and Dick B. , Femtosecond Laser-Assisted Cataract Surgery in Pediatric Marfan Syndrome, Journal of Refractive Surgery. (2013) 29, no. 9, 650–652, 10.3928/1081597X-20130819-06, 24016350. 24016350

[10] Taravella M. J. , Meghpara B. , Frank G. , Gensheimer W. , and Davidson R. , Femtosecond Laser-Assisted Cataract Surgery in Complex Cases, Journal of Cataract and Refractive Surgery. (2016) 42, no. 6, 813–816, 10.1016/j.jcrs.2016.02.049, 27373386. 27373386

[11] Hwang E. S. , Perez-Straziota C. E. , Kim S. W. , Santhiago M. R. , and Randleman J. B. , Distinguishing Highly Asymmetric Keratoconus Eyes Using Combined Scheimpflug and Spectral-Domain OCT Analysis, Ophthalmology. (2018) 125, no. 12, 1862–1871, 10.1016/j.ophtha.2018.06.020, 30055838. 30055838 PMC6246819

[12] Ali M. H. , Javaid M. , Jamal S. , and Butt N. H. , Femtosecond Laser Assisted Cataract Surgery, Beginning of a New Era in Cataract Surgery, Oman Journal of Ophthalmology. (2015) 8, no. 3, 141–146, 10.4103/0974-620X.169892, 26903717. 26903717 PMC4738656

